# Burden of comorbidities in people with multiple sclerosis: a population-based study in Catalonia

**DOI:** 10.3389/fneur.2025.1699641

**Published:** 2025-12-11

**Authors:** Toni Mora

**Affiliations:** School of Economics & Social Sciences, Universitat Internacional de Catalunya, Barcelona, Spain

**Keywords:** multiple sclerosis, comorbidity, population-based studies, administrative data, epidemiology, sex characteristics

## Abstract

**Background:**

People with multiple sclerosis (MS) often present with chronic comorbidities that complicate care and affect prognosis. Population-based evidence in Europe is limited.

**Objective:**

To describe the prevalence of selected comorbidities among individuals with MS in Catalonia, Spain, using administrative health records.

**Methods:**

We analysed data from 9,998 people diagnosed with MS (2013–2017), including demographic and diagnostic information across all care levels. Prevalence estimates for 23 comorbidities were stratified by age and sex, and, where available, compared with those of the general population.

**Results:**

The MS population had a higher prevalence of psychiatric, metabolic, and autoimmune comorbidities in all age groups. Young women (18–40 years) showed increased rates of depression, anxiety, thyroid disease, and migraine, while middle-aged men (50–60 years) had higher rates of hypertension, hyperlipidaemia, and diabetes.

**Conclusion:**

MS is associated with a substantial, early-onset comorbidity burden, underscoring the need for integrated, multidisciplinary care across the life course.

## Introduction

Multiple sclerosis (MS) is a chronic, immune-mediated demyelinating disorder of the central nervous system, typically diagnosed in early adulthood with progressive disability. The clinical course of MS is highly variable, and its impact extends beyond neurological impairment (1–3).

Increasing evidence suggests that individuals with MS are more likely to experience multiple comorbidities compared to the general population. These comorbid conditions may influence progression, treatment choices, and quality of life (4, 5). In particular, comorbidity has been associated with delayed diagnosis, increased relapse rates, worse functional outcomes, and higher healthcare resource utilisation.

Despite its relevance, comprehensive population-level data on the prevalence of comorbidities in MS remain limited, especially in European contexts (6). Many studies rely on clinical cohorts or insurance data, underrepresenting some groups.

In this study, we examine the burden of comorbidities among individuals diagnosed with MS in Catalonia, Spain, using a large, adult population-based administrative dataset. By leveraging linked data across healthcare settings, we aim to provide robust epidemiological estimates of selected chronic conditions in the MS population, stratified by age and gender, and to compare these patterns with the general population where feasible.

## Patients and methods

### Study design and data source

We conducted a population-based, cross-sectional study using administrative health records from the Agency for Health Quality and Assessment of Catalonia (AQuAS). The database consolidates information from the public healthcare system of Catalonia, encompassing all adults diagnosed with MS between 2013 and 2017 (9,998 individuals). Therefore, the study cohort represents virtually the entire adult population with MS in Catalonia receiving care within the national health system.

The dataset includes linked records across primary care, hospital admissions, and emergency departments, using anonymised identifiers. Variables included visit dates, diagnoses (coded using ICD-9, extracted 2019), age, sex, and drug copayment status. ICD-9 coding was used throughout, as this was the system provided by AQuAS at the time; no recoding to ICD-10 was performed. All records were harmonised across care levels (primary care, hospital, emergency). Duplicate diagnoses were removed, and prevalence was calculated using aggregated unique identifiers per patient and ICD-9 category. The study received approval from the Ethical Review Board at Hospital Clínic de Barcelona.

The Catalan Health Service provides near-universal coverage, and even individuals partially managed in private settings are included in the public system, as disease-modifying therapies and chronic medications are dispensed and co-financed through public pharmacies. This ensures that nearly all MS patients in Catalonia are captured within the AQuAS database, minimising selection bias. However, the dataset does not include certain clinical variables, such as MS phenotype, disease duration, disability level (as measured by EDSS), or detailed treatment information. The absence of these parameters limits the ability to explore how disease characteristics influence comorbidity patterns. Moreover, as the design is cross-sectional, the temporal sequence between MS and comorbidities cannot be established, and causal inference is therefore precluded. Because diagnoses were captured cross-sectionally over 2013–2017, the temporal sequence between MS onset and comorbidity diagnosis could not be determined.

### Study population

We included all individuals who received a diagnosis of MS (ICD-9 code 340). For population-level comparisons, AQuAS provided aggregated demographic data related to the adult prevalence of each selected comorbidity from the general Catalan population for the same period (6,155,980 individuals), stratified by age according to clinicians’ indications from the Hospital Clinic de Barcelona, sex, and copayment categories (7). The same ICD-9 coding system and study period were used for both MS patients and the general population, ensuring comparability.

### Comorbidities

Twenty-three chronic comorbidities were selected *a priori* by clinicians based on clinical relevance and prevalence in prior studies. These included psychiatric conditions (e.g., anxiety, depression), cardiovascular and metabolic diseases (e.g., hypertension, diabetes, hyperlipidaemia), autoimmune disorders, and other neurologically or systemically relevant conditions. Diagnoses were identified across all care levels using ICD-9 codes, capturing both primary and secondary diagnostic positions. The cancer category included ICD-9 codes for malignant neoplasms as well as some benign and uncertain behaviour neoplasms, which may partly explain the higher prevalence observed. Diabetes consists of both type 1 and type 2 forms. The complete list of ICD-9 codes is provided in Supplementary Table S1.

### Statistical analysis

We calculated the prevalence of each comorbidity per 10,000 inhabitants among individuals with MS and compared these estimates to those from the general population. Prevalence was reported overall and stratified by age group and sex. Descriptive statistics were used to characterise the study population, and we report the main differences in the prevalence of comorbidities in the results section. Further comparative or regression analyses were not performed, as the available datasets did not allow such analyses. Given the cross-sectional design of the study and the nature of the available data, no causal relationships between multiple sclerosis and the observed comorbidities can be inferred. Formal tests of difference in prevalence between the MS and general population were conducted, and all reported comparisons reached statistical significance (*p* < 0.05). Therefore, significance markers were omitted from the figures for clarity. Confidence intervals were not displayed in the statistics to improve visual clarity, but are available upon request.

## Results

Across all groups, individuals with MS consistently exhibited a higher prevalence of psychiatric, metabolic, and autoimmune conditions. This pattern was evident irrespective of age or sex, indicating that the elevated comorbidity burden associated with MS is not confined to specific population segments. A full summary of prevalence estimates for all 23 comorbidities by age and sex is provided in Supplementary Table S1.

The age and sex distribution of the MS population was strongly skewed toward women (68.5%), with most cases occurring between ages 40 and 60 (Supplementary Figure S0). The crude prevalence of MS in the population was 13.3 per 10,000 population, with a female-to-male ratio of more than 2:1.

**Figure 1:**
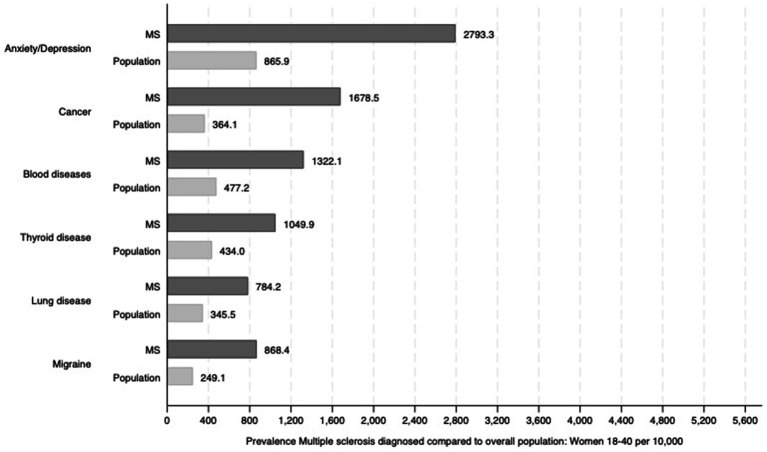
Prevalence of selected comorbidities among women aged 18–40 years diagnosed with MS, compared to the general female population in the same age group. We report those statistically significant differences (*p* < 0.05).

The burden of comorbidities varied markedly by sex and age. In women aged 18–40 years, the prevalence of depression and anxiety was 2,793.3 per 10,000, compared to 865.9 per 10,000 in general females. Cancer was the second most common comorbidity in this group, with 1,678.5 cases per 10,000 among those with MS versus 364.1 per 10,000 in the general female population. Other notable conditions included thyroid disease (1,049.9 per 10,000), blood disorders (1,322.1 per 10,000), and migraine (868.4 per 10,000) (Figure 1). These patterns indicate an elevated burden from early adulthood.

**Figure 2:**
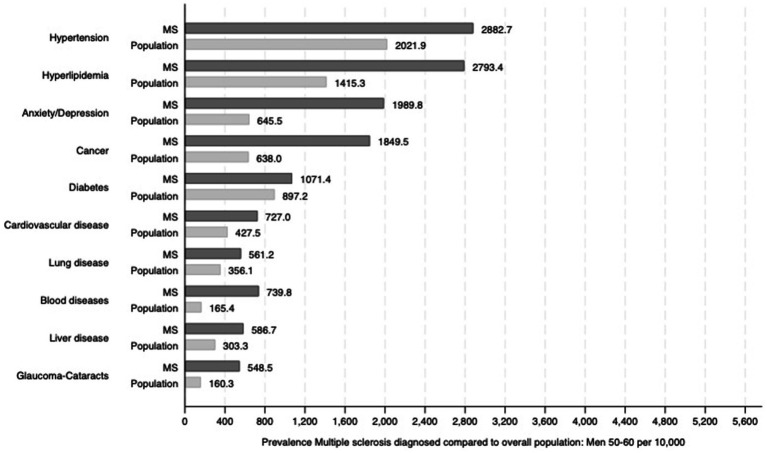
Prevalence of selected comorbidities among men aged 50–60 years diagnosed with MS, compared to the general male population in the same age group. We report statistically significant differences (*p* < 0.05).

Among men aged 50–60 years, hypertension (2,882.7 per 10,000), hyperlipidaemia (2,793.4 per 10,000), and depression/anxiety (1,989.8 per 10,000) were the most common comorbidities, all higher than in general males. Cancer was also notably prevalent in this group (1,849.5 per 10,000 vs. 638.0 per 10,000 in the general population). Diabetes and cardiovascular disease also increased with age (Figure 2).

## Discussion

This population-based study provides new evidence on the burden of comorbidities among individuals with MS in Catalonia. Our findings show that, compared to the general population, people with MS exhibit a substantially higher prevalence of psychiatric (e.g., depression, anxiety), endocrine (e.g., thyroid disorders, diabetes), and autoimmune (e.g., inflammatory bowel disease, rheumatoid arthritis) conditions across all age groups, with marked differences among women. For example, the prevalence of depression in women aged 30–44 years with MS was more than three times higher than in their peers without MS, and the prevalence of thyroid disorders was nearly doubled. These patterns were consistent across other chronic conditions, confirming the burden is not limited to older age.

These results have clinical and public health implications. The high rates of depression, anxiety, and other chronic conditions observed even in younger adults underscore the need for early and proactive management of multimorbidity in MS care. Comprehensive, multidisciplinary approaches should be considered as part of routine follow-up, particularly for women who may experience a greater cumulative burden of disease. This study informs healthcare planning in Spain by quantifying the early and persistent burden of comorbidities in people with MS using real-world, population-level data. These findings may inform national strategies to promote integrated care models that address both neurological and non-neurological health needs across the lifespan.

Our study builds on an earlier population-based, case–control study in Catalonia (8) that reported elevated odds of specific comorbidities in people with MS—such as stroke, epilepsy, bipolar disorder, and depression. While their research focused on a narrower set of conditions, our analysis extends this by including a broader spectrum of 23 comorbidities (psychiatric, metabolic, and autoimmune) and providing prevalence measures stratified by age and sex. Notably, we quantify differences such as a tripled rate of depression in young women and a twofold increase in thyroid disease across all age groups. By doing so, we provide a more comprehensive depiction of the comorbidity burden, informing more targeted and equitable interventions and health resource planning. Some apparent discrepancies, such as the comparatively low prevalence of migraine and the high prevalence of certain systemic conditions, likely reflect coding and grouping variability within administrative data. Less disabling or less frequently coded conditions, such as primary headaches, may be underrepresented compared to chronic or severe diagnoses.

Although most comorbidities were more frequent among people with MS, some conditions appeared less common, possibly reflecting differential diagnostic attention or coding practices within neurological care. The observed sex- and age-specific patterns may reflect both biological susceptibility and differences in healthcare utilisation, including more frequent contact with health services among women. This study is strengthened by the use of large-scale administrative data covering the entire publicly insured population of Catalonia. Nonetheless, some limitations apply. Diagnoses were based on ICD-9 coding from administrative records and may underreport conditions that are less frequently coded. Diagnoses may be underreported or inaccurately coded, which could affect prevalence estimates. Additionally, mild or asymptomatic conditions may remain underdiagnosed in administrative records, particularly when they do not require specialised care. Moreover, MS patients are more closely monitored and undergo more tests, which may result in a more comprehensive recording of comorbidities compared to the general population. MS patients exhibit higher attendance rates compared to the general population. However, given the near-universal nature of the Catalan public health system and the inclusion of patients co-financed for disease-modifying therapies, underrepresentation of individuals followed partly in private settings is unlikely. To strengthen the evidence base, future research should adopt longitudinal designs, control for confounding variables, evaluate relevant clinical outcomes, and explore the mechanisms underlying these associations, as well as their long-term impact on health and services (9). Additionally, the dataset lacked information on relevant potential confounders such as body mass index, disability status, medication use, or lifestyle factors, which limits the capacity for adjusted analyses and causal interpretation. Although the dataset included all identified MS patients over a five-year period, the present analysis focused on aggregated prevalence rather than individual trajectories; therefore, causal relationships cannot be established.

## Conclusion

People with multiple sclerosis (MS) frequently present comorbid chronic conditions that may influence diagnosis, prognosis, and treatment. Most evidence on comorbidity in MS comes from selected samples or registries, with limited population coverage. This study quantifies the early and persistent burden of psychiatric, metabolic, and autoimmune comorbidities in a whole-population MS cohort, highlighting marked sex- and age-specific differences. It highlights sex- and age-specific differences, with an early burden of mental health conditions that persists throughout the life course. This study provides population-wide evidence on the burden of psychiatric, metabolic, and autoimmune comorbidities among MS patients using administrative health data.

## Data Availability

The datasets analyzed during the current study are not publicly available due to legal restrictions. The datasets are based on administrative health registers owned by the regional public administration of Catalonia and the necessary authorization was obtained for data access and analysis. Requests to access the datasets should be directed to TM, tmora@uic.es.
