# Supplementary material for: Burden of comorbidities in people with multiple sclerosis: a population-based study in Catalonia
Source: Front Neurol. 2025 Dec 11;16:1699641. doi: 10.3389/fneur.2025.1699641 (PMC12738334; doi:10.3389/fneur.2025.1699641)
Supplement: Supplementary file 1 [file Supplementary_file_1.docx]

**Supplementary**

**Table S1. Prevalence (per 10,000 inhabitants) of 23 clinically relevant comorbidities among adults with multiple sclerosis in Catalonia, stratified by age group and sex (2013–2017).**

|  | **Men** | | | | **Women** | | | |
| --- | --- | --- | --- | --- | --- | --- | --- | --- |
|  | **[18-40[** | **[40-50[** | **[50-60[** | **[60-]** | **[18-40[** | **[40-50[** | **[50-60[** | **[60-]** |
| Anxiety/Depression | 1804.7 | 2009.0 | 1989.8 | 1373.9 | 2793.3 | 3082.5 | 2954.5 | 2465.9 |
| Arthrosis | 133.1 | 203.2 | 446.4 | 1024.8 | 77.8 | 290.6 | 861.2 | 2119.3 |
| Autoimmune diseases | 133.1 | 203.2 | 127.6 | 315.3 | 272.2 | 326.9 | 412.7 | 522.7 |
| Blood diseases | 192.3 | 338.6 | 739.8 | 1475.2 | 1322.1 | 1956.4 | 1967.7 | 1909.1 |
| Cancer | 1153.8 | 1241.5 | 1849.5 | 2939.2 | 1678.5 | 2179.6 | 2667.5 | 2789.8 |
| Cardiovascular disease | 88.8 | 338.6 | 727.0 | 1723.0 | 155.5 | 275.0 | 496.4 | 943.2 |
| Diabetes | 162.7 | 507.9 | 1071.4 | 1982.0 | 136.1 | 285.4 | 556.2 | 1625.0 |
| Epilepsy | 384.6 | 361.2 | 293.4 | 304.1 | 213.9 | 275.0 | 245.2 | 358.0 |
| Fibromyalgia | 162.7 | 304.7 | 191.3 | 225.2 | 395.3 | 591.6 | 789.5 | 573.9 |
| Glaucoma-Cataracts | 74.0 | 225.7 | 548.5 | 1610.4 | 58.3 | 249.1 | 568.2 | 1778.4 |
| Hyperlipidaemia | 576.9 | 1647.9 | 2793.4 | 3479.7 | 427.7 | 944.5 | 2308.6 | 3448.9 |
| Hypertension | 236.7 | 1219.0 | 2882.7 | 4977.5 | 226.8 | 819.9 | 2171.1 | 4670.5 |
| Irritable bowel syndrome | 0.0 | 33.9 | 25.5 | 11.3 | 19.4 | 36.3 | 17.9 | 22.7 |
| Liver disease | 162.7 | 406.3 | 586.7 | 653.2 | 123.1 | 243.9 | 436.6 | 590.9 |
| Lung disease | 636.1 | 632.1 | 561.2 | 1171.2 | 784.2 | 731.7 | 747.6 | 869.3 |
| Migraine | 473.4 | 259.6 | 140.3 | 56.3 | 868.4 | 877.0 | 622.0 | 323.9 |
| Osteoporosis | 74.0 | 101.6 | 255.1 | 439.2 | 51.8 | 207.6 | 831.3 | 2460.2 |
| Other psychiatric: bipolar, schizophrenia | 147.9 | 169.3 | 127.6 | 123.9 | 84.3 | 109.0 | 143.5 | 108.0 |
| Peptic ulcer disease | 14.8 | 79.0 | 127.6 | 214.0 | 25.9 | 46.7 | 35.9 | 153.4 |
| Renal failure | 59.2 | 169.3 | 318.9 | 1103.6 | 51.8 | 51.9 | 245.2 | 823.9 |
| Thyroid disease | 192.3 | 349.9 | 459.2 | 518.0 | 1049.9 | 1328.5 | 2015.6 | 1965.9 |
| Vit B12 deficiency (+ folate) | 133.1 | 180.6 | 204.1 | 326.6 | 155.5 | 176.4 | 263.2 | 329.5 |

Note: Prevalence estimates are expressed per 10,000 inhabitants. Comorbidities were defined using ICD-9 diagnostic codes identified across all care levels (primary care, hospital admissions, and emergency visits). The same coding and period were used for the comparison with the general population. Age categories: 18–39, 40–49, 50–59, and ≥60 years. Data source: Agency for Health Quality and Assessment of Catalonia (AQuAS).

**Figure S0. Age and sex distribution of individuals diagnosed with MS in Catalonia between 2013 and 2017.**

**
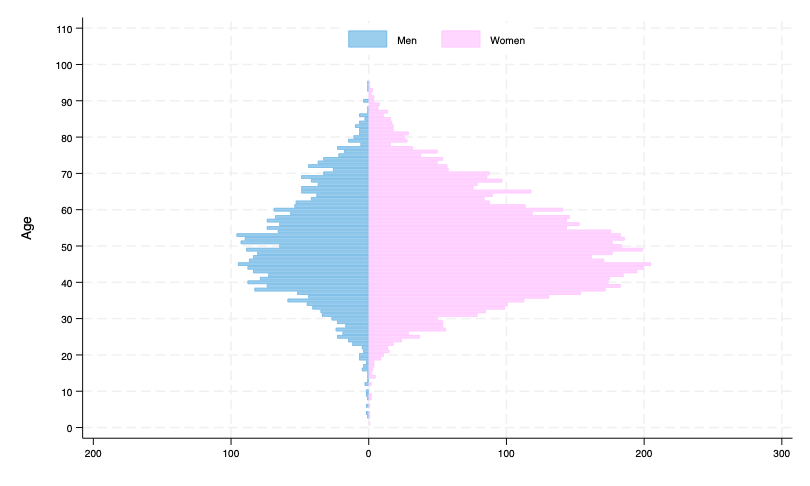
**

**Figure S1. Prevalence of selected comorbidities among men aged 18–40 years diagnosed with MS, compared to the general male population in the same age group. We report statistically significant differences (p < 0.05).**


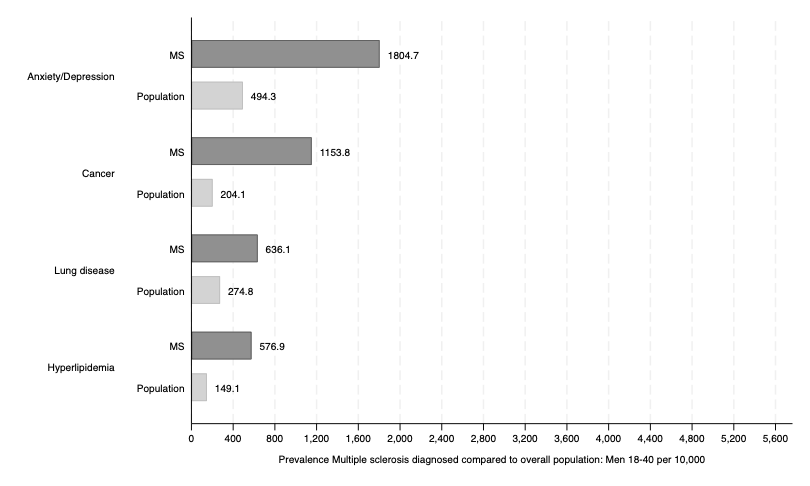


**Figure S2. Prevalence of selected comorbidities among men aged 40–50 years diagnosed with MS, compared to the general male population in the same age group. We report statistically significant differences (p < 0.05).**


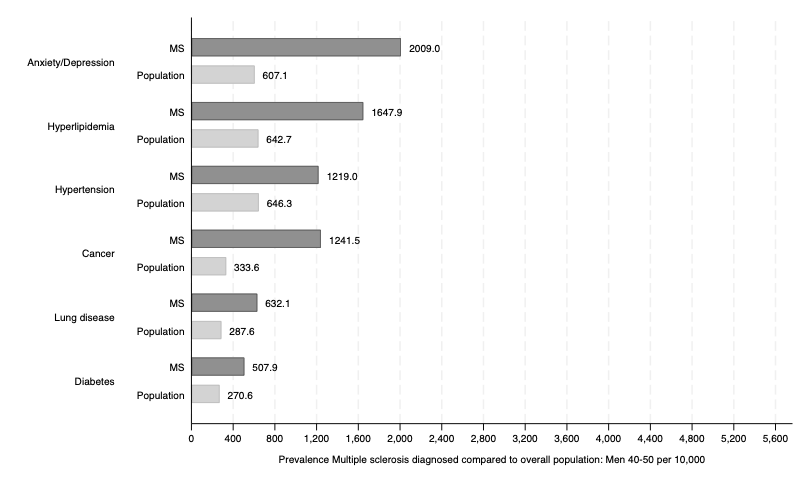


**Figure S3. Prevalence of selected comorbidities among women aged 40–50 years diagnosed with MS, compared to the general female population in the same age group. We report statistically significant differences (p < 0.05).**


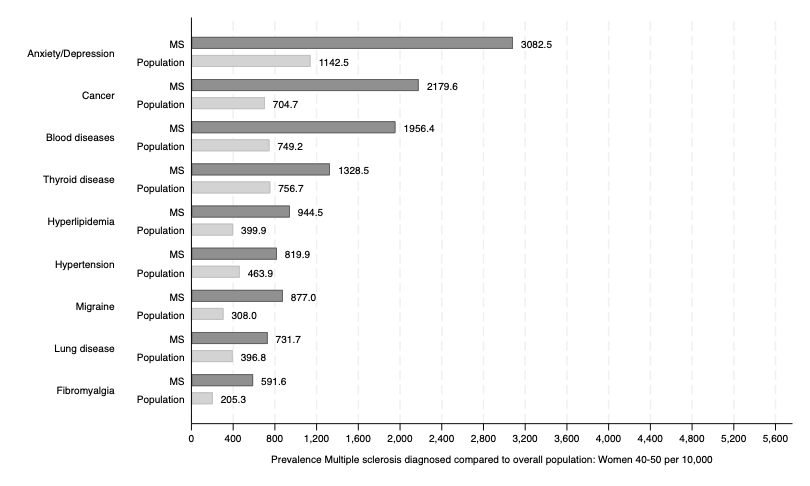


**Figure S4. Prevalence of selected comorbidities among women aged 50–60 years diagnosed with MS, compared to the general female population in the same age group. We report statistically significant differences (p < 0.05).**


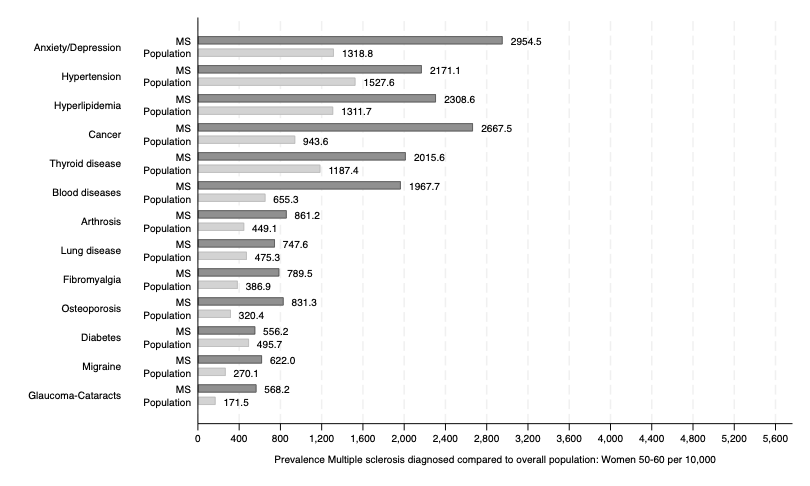


**Figure S5. Prevalence of selected comorbidities among men aged over 60 years diagnosed with MS, compared to the general male population in the same age group. We report statistically significant differences (p < 0.05).**


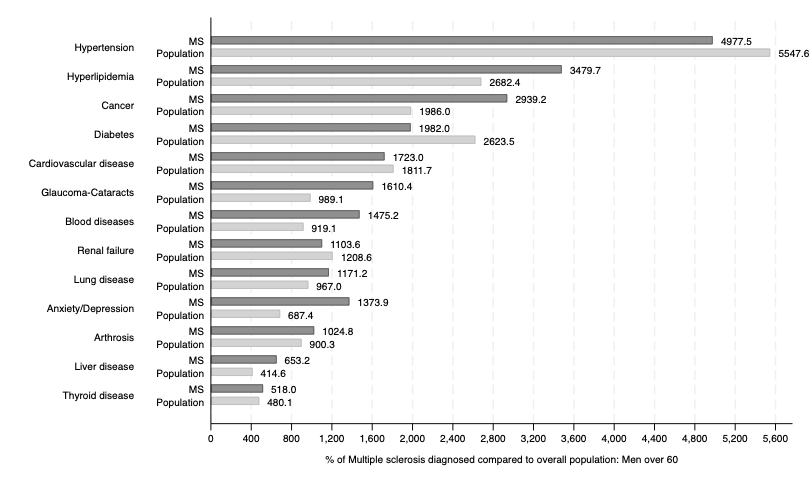


**Figure S6. Prevalence of selected comorbidities among women aged over 60 years diagnosed with MS, compared to the general female population in the same age group. We report statistically significant differences (p < 0.05).**


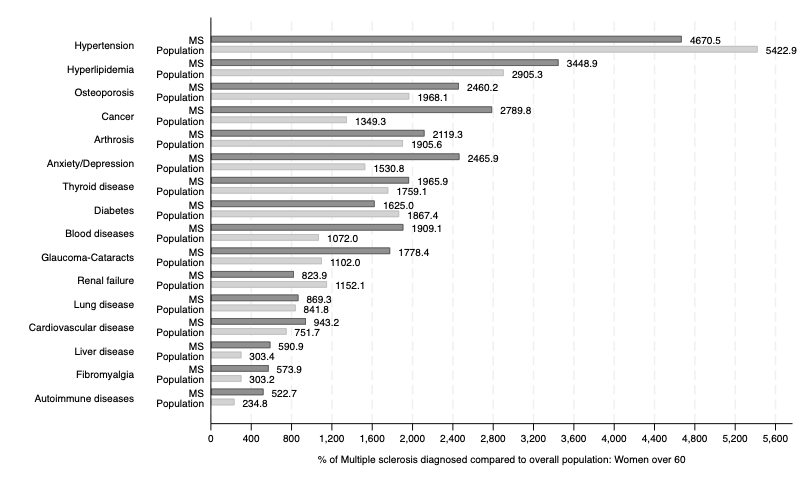


**Table ICD-9 codes for comorbidities.**

| **Other psyquiatric: bipolar, squizophrenia** | **Anxiety** | **Depression** | **Epilepsy** | **Irritable bowel**  **syndrome** | **Liver**  **disease** | **Peptic ulcer**  **disease** | **Renal**  **failure** | **Blood**  **diseases** | **Fibromyalgia** |
| --- | --- | --- | --- | --- | --- | --- | --- | --- | --- |
| 295  2950  29500  29501  29502  29503  29504  29505  2951  29510  29511  29512  29513  29514  29515  2952  29520  29521  29522  29523  29524  29525  2953  29530  29531  29532  29533  29534  29535  2954  29540  29541  29542  29543  29544  29545  2955  29550  29551  29552  29553  29554  29555  2956  29560  29561  29562  29563  29564  29565  2957  29570  29571  29572  29573  29574  29575  296  2960  29600  29601  29602  29603  29604  29605  29606  2961  29610  29611  29612  29613  29614  29615  29616  2964  29640  29641  29642  29643  29644  29645  29646  2965  29650  29651  29652  29653  29654  29655  29656  2966  29660  29661  29662  29663  29664  29665  29666  2967  2968  29680  29681  29682 | 300  3000  30000  30001  30002  30009  30921  30924  30928 | 2962  29620  29621  29622  29623  29624  29625  29626  2963  29630  29631  29632  29633  29634  29635  29636  311 | 345  3450  34500  34501  3451  34510  34511  3452  3453  3454  34540  34541  3455  34550  34551  3456  34560  34561  3457  34570  34571  3458  34580  34581  3459  34590  34591 | 7879  78799 | 570  571  5710  5711  5712  5713  5714  57140  57141  57142  57149  5715  5716  5718  5719  572  5720  5721  5722  5723  5724  5728  573  5730  5731  5732  5733  5734  5735  5738  5739 | 531  5310  53100  53101  5311  53110  53111  5312  53120  53121  5313  53130  53131  5314  53140  53141  5315  53150  53151  5316  53160  53161  5317  53170  53171  5319  53190  53191  532  5320  53200  53201  5321  53210  53211  5322  53220  53221  5323  53230  53231  5324  53240  53241  5325  53250  53251  5326  53260  53261  5327  53270  53271  5329  53290  53291  533  5330  53300  53301  5331  53310  53311  5332  53320  53321  5333  53330  53331  5334  53340  53341  5335  53350  53351  5336  53360  53361  5337  53370  53371  5339  53390  53391  534  5340  53400  53401  5341  53410  53411  5342  53420  53421  5343  53430  53431  5344  53440  53441  5345  53450  53451  5346  53460  53461  5347  53470  53471  5349  53490  53491 | 584  5845  5846  5847  5848  5849  585  5851  5852  5853  5854  5855  5856  5859  586 | 280  2800  2801  2808  2809  281  2810  2811  2812  2813  2814  2818  2819  282  2820  2821  2822  2823  2824  28240  28241  28242  28243  28244  28245  28246  28247  28249  2825  2826  28260  28261  28262  28263  28264  28268  28269  2827  2828  2829  283  2830  2831  28310  28311  28319  2832  2839  284  2840  28401  28409  2841  28411  28412  28419  2842  2848  28481  28489  2849  285  2850  2851  2852  28521  28522  28529  2853  2858  2859  286  2860  2861  2862  2863  2864  2865  28652  28653  28659  2866  2867  2869 | 7291 |

**Contd.**

| **Osteoporosis** | **Arthrosis** | **Autoimmune**  **diseases** | **Vit B12 deficiency (+ folate)** | **Thyroid**  **disease** | **Migraine** | **Glaucoma-Cataracts** | **Diabetes** | **Lung**  **disease** | **Cardiovascular**  **disease** | **Hypertension** | **Hyperlypidemia** |
| --- | --- | --- | --- | --- | --- | --- | --- | --- | --- | --- | --- |
| 7330  73300  73301  73302  73303  73309  7331  73310  73311  73312  73313  73314  73315  73316  73319 | 715  7150  71500  71504  71509  7151  71510  71511  71512  71513  71514  71515  71516  71517  71518  7152  71520  71521  71522  71523  71524  71525  71526  71527  71528  7153  71530  71531  71532  71533  71534  71535  71536  71537  71538  7158  71580  71589  7159  71590  71591  71592  71593  71594  71595  71596  71597  71598 | 3640  3643  7100  7102  714  7140  7141  7142  7143  555  5550  5551  5552  5559  556  5560  5561  5562  5563  5564  5565  5566  5568  5569 | 2662 | 240  2400  2409  241  2410  2411  2419  242  2420  24200  24201  2421  24210  24211  2422  24220  24221  2423  24230  24231  2424  24240  24241  2428  24280  24281  2429  24290  24291  243  244  2440  2441  2442  2443  2448  2449  245  2450  2451  2452  2453  2454  2458  2459  246  2460  2461  2462  2463  2468  2469 | 346  3460  34600  34601  34602  34603  3461  34610  34611  34612  34613  3462  34620  34621  34622  34623  3463  34630  34631  34632  34633  3464  34640  34641  34642  34643  3465  34650  34651  34652  34653  3466  34660  34661  34662  34663  3467  34670  34671  34672  34673  3468  34680  34681  34682  34683  3469  34690  34691  34692  34693 | 365  3650  36500  36501  36502  36503  36504  36505  36506  3651  36510  36511  36512  36513  36514  36515  3652  36520  36521  36522  36523  36524  3653  36531  36532  3654  36541  36542  36543  36544  3655  36551  36552  36559  3656  36560  36561  36562  36563  36564  36565  3657  36570  36571  36572  36573  36574  3658  36581  36582  36583  36589  3659  366  3660  36600  36601  36602  36603  36604  36609  3661  36610  36611  36612  36613  36614  36615  36616  36617  36618  36619  3662  36620  36621  36622  36623  3663  36630  36631  36632  36633  36634  3664  36641  36642  36643  36644  36645  36646  3665  36650  36651  36652  36653  3668  3669 | 249  2490  24900  24901  2491  24910  24911  2492  24920  24921  2493  24930  24931  2494  24940  24941  2495  24950  24951  2496  24960  24961  2497  24970  24971  2498  24980  24981  2499  24990  24991  250  2500  25000  25001  25002  25003  2501  25010  25011  25012  25013  2502  25020  25021  25022  25023  2503  25030  25031  25032  25033  2504  25040  25041  25042  25043  2505  25050  25051  25052  25053  2506  25060  25061  25062  25063  2507  25070  25071  25072  25073  2508  25080  25081  25082  25083  2509  25090  25091  25092  25093 | 491  4910  4911  4912  49120  49121  49122  4918  4919  492  4920  4928  493  4930  49300  49301  49302  4931  49310  49311  49312  4932  49320  49321  49322  4938  49381  49382  4939  49390  49391  49392 | 410  4100  41000  41001  41002  4101  41010  41011  41012  4102  41020  41021  41022  4103  41030  41031  41032  4104  41040  41041  41042  4105  41050  41051  41052  4106  41060  41061  41062  4107  41070  41071  41072  4108  41080  41081  41082  4109  41090  41091  41092  411  4110  4111  4118  41181  41189  412  413  4130  4131  4139  414  4140  41400  41401  41402  41403  41404  41405  41406  41407  4141  41410  41411  41412  41419  4142  4143  4144  4148  4149  440  4400  4401  4402  44020  44021  44022  44023  44024  44029  4403  44030  44031  44032  4404  4408  4409  441  4410  44100  44101  44102  44103  4411  4412  4413  4414  4415  4416  4417  4419  442  4420  4421  4422  4423  4428  44281  44282  44283  44284  44289  4429  443  4430  4431  4432  44321  44322  44323  44324  44329  4438  44381  44382  44389  4439  444  4440  44401  44409  4441  4442  44421  44422  4448  44481  44489  4449  445  4450  44501  44502  4458  44581  44589 | 401  4010  4011  4019  402  4020  40200  40201  4021  40210  40211  4029  40290  40291  403  4030  40300  40301  4031  40310  40311  4039  40390  40391  404  4040  40400  40401  40402  40403  4041  40410  40411  40412  40413  4049  40490  40491  40492  40493  405  4050  40501  40509  4051  40511  40519  4059  40591  40599 | 272  2720  2721  2722  2723  2724 |

**ICD-codes for Cancer**: 140 1400 1401 1403 1404 1405 1406 1408 1409 141 1410 1411 1412 1413 1414 1415 1416 1418 1419 142 1420 1421 1422 1428 1429 143 1430 1431 1438 1439 144 1440 1441 1448 1449 145 1450 1451 1452 1453 1454 1455 1456 1458 1459 146 1460 1461 1462 1463 1464 1465 1466 1467 1468 1469 147 1470 1471 1472 1473 1478 1479 148 1480 1481 1482 1483 1488 1489 149 1490 1491 1498 1499 150 1500 1501 1502 1503 1504 1505 1508 1509 151 1510 1511 1512 1513 1514 1515 1516 1518 1519 152 1520 1521 1522 1523 1528 1529 153 1530 1531 1532 1533 1534 1535 1536 1537 1538 1539 154 1540 1541 1542 1543 1548 155 1550 1551 1552 156 1560 1561 1562 1568 1569 157 1570 1571 1572 1573 1574 1578 1579 158 1580 1588 1589 159 1590 1591 1598 1599 160 1600 1601 1602 1603 1604 1605 1608 1609 161 1610 1611 1612 1613 1618 1619 162 1620 1622 1623 1624 1625 1628 1629 163 1630 1631 1638 1639 164 1640 1641 1642 1643 1648 1649 165 1650 1658 1659 170 1700 1701 1702 1703 1704 1705 1706 1707 1708 1709 171 1710 1712 1713 1714 1715 1716 1717 1718 1719 172 1720 1721 1722 1723 1724 1725 1726 1727 1728 1729 173 1730 17300 17301 17302 17309 1731 17310 17311 17312 17319 1732 17320 17321 17322 17329 1733 17330 17331 17332 17339 1734 17340 17341 17342 17349 1735 17350 17351 17352 17359 1736 17360 17361 17362 17369 1737 17370 17371 17372 17379 1738 17380 17381 17382 17389 1739 17390 17391 17392 17399 174 1740 1741 1742 1743 1744 1745 1746 1748 1749 175 1750 1759 176 1760 1761 1762 1763 1764 1765 1768 1769 179 180 1800 1801 1808 1809 181 182 1820 1821 1828 183 1830 1832 1833 1834 1835 1838 1839 184 1840 1841 1842 1843 1844 1848 1849 185 186 1860 1869 187 1871 1872 1873 1874 1875 1876 1877 1878 1879 188 1880 1881 1882 1883 1884 1885 1886 1887 1888 1889 189 1890 1891 1892 1893 1894 1898 1899 190 1900 1901 1902 1903 1904 1905 1906 1907 1908 1909 191 1910 1911 1912 1913 1914 1915 1916 1917 1918 1919 192 1920 1921 1922 1923 1928 1929 193 194 1940 1941 1943 1944 1945 1946 1948 1949 195 1950 1951 1952 1953 1954 1955 1958 196 1960 1961 1962 1963 1965 1966 1968 1969 197 1970 1971 1972 1973 1974 1975 1976 1977 1978 198 1980 1981 1982 1983 1984 1985 1986 1987 1988 19881 19882 19889 199 1990 1991 1992 200 2000 20000 20001 20002 20003 20004 20005 20006 20007 20008 2001 20010 20011 20012 20013 20014 20015 20016 20017 20018 2002 20020 20021 20022 20023 20024 20025 20026 20027 20028 2003 20030 20031 20032 20033 20034 20035 20036 20037 20038 2004 20040 20041 20042 20043 20044 20045 20046 20047 20048 2005 20050 20051 20052 20053 20054 20055 20056 20057 20058 2006 20060 20061 20062 20063 20064 20065 20066 20067 20068 2007 20070 20071 20072 20073 20074 20075 20076 20077 20078 2008 20080 20081 20082 20083 20084 20085 20086 20087 20088 201 2010 20100 20101 20102 20103 20104 20105 20106 20107 20108 2011 20110 20111 20112 20113 20114 20115 20116 20117 20118 2012 20120 20121 20122 20123 20124 20125 20126 20127 20128 2014 20140 20141 20142 20143 20144 20145 20146 20147 20148 2015 20150 20151 20152 20153 20154 20155 20156 20157 20158 2016 20160 20161 20162 20163 20164 20165 20166 20167 20168 2017 20170 20171 20172 20173 20174 20175 20176 20177 20178 2019 20190 20191 20192 20193 20194 20195 20196 20197 20198 202 2020 20200 20201 20202 20203 20204 20205 20206 20207 20208 2021 20210 20211 20212 20213 20214 20215 20216 20217 20218 2022 20220 20221 20222 20223 20224 20225 20226 20227 20228 2023 20230 20231 20232 20233 20234 20235 20236 20237 20238 2024 20240 20241 20242 20243 20244 20245 20246 20247 20248 2025 20250 20251 20252 20253 20254 20255 20256 20257 20258 2026 20260 20261 20262 20263 20264 20265 20266 20267 20268 2027 20270 20271 20272 20273 20274 20275 20276 20277 20278 2028 20280 20281 20282 20283 20284 20285 20286 20287 20288 2029 20290 20291 20292 20293 20294 20295 20296 20297 20298 203 2030 20300 20301 20302 2031 20310 20311 20312 2038 20380 20381 20382 204 2040 20400 20401 20402 2041 20410 20411 20412 2042 20420 20421 20422 2048 20480 20481 20482 2049 20490 20491 20492 205 2050 20500 20501 20502 2051 20510 20511 20512 2052 20520 20521 20522 2053 20530 20531 20532 2058 20580 20581 20582 2059 20590 20591 20592 206 2060 20600 20601 20602 2061 20610 20611 20612 2062 20620 20621 20622 2068 20680 20681 20682 2069 20690 20691 20692 207 2070 20700 20701 20702 2071 20710 20711 20712 2072 20720 20721 20722 2078 20780 20781 20782 208 2080 20800 20801 20802 2081 20810 20811 20812 2082 20820 20821 20822 2088 20880 20881 20882 2089 20890 20891 20892 209 2090 20900 20901 20902 20903 2091 20910 20911 20912 20913 20914 20915 20916 20917 2092 20920 20921 20922 20923 20924 20925 20926 20927 20929 2093 20930 20931 20932 20933 20934 20935 20936 2094 20940 20941 20942 20943 2095 20950 20951 20952 20953 20954 20955 20956 20957 2096 20960 20961 20962 20963 20964 20965 20966 20967 20969 2097 20970 20971 20972 20973 20974 20975 20979 210 2100 2101 2102 2103 2104 2105 2106 2107 2108 2109 211 2110 2111 2112 2113 2114 2115 2116 2117 2118 2119 212 2120 2121 2122 2123 2124 2125 2126 2127 2128 2129 213 2130 2131 2132 2133 2134 2135 2136 2137 2138 2139 214 2140 2141 2142 2143 2144 2148 2149 215 2150 2152 2153 2154 2155 2156 2157 2158 2159 216 2160 2161 2162 2163 2164 2165 2166 2167 2168 2169 217 218 2180 2181 2182 2189 219 2190 2191 2198 2199 220 221 2210 2211 2212 2218 2219 222 2220 2221 2222 2223 2224 2228 2229 223 2230 2231 2232 2233 2238 22381 22389 2239 224 2240 2241 2242 2243 2244 2245 2246 2247 2248 2249 225 2250 2251 2252 2253 2254 2258 2259 226 227 2270 2271 2273 2274 2275 2276 2278 2279 228 2280 22800 22801 22802 22803 22804 22809 2281 229 2290 2298 2299 230 2300 2301 2302 2303 2304 2305 2306 2307 2308 2309 231 2310 2311 2312 2318 2319 232 2320 2321 2322 2323 2324 2325 2326 2327 2328 2329 233 2330 2331 2332 2333 23330 23331 23332 23339 2334 2335 2336 2337 2339 234 2340 2348 2349 235 2350 2351 2352 2353 2354 2355 2356 2357 2358 2359 236 2360 2361 2362 2363 2364 2365 2366 2367 2369 23690 23691 23699 237 2370 2371 2372 2373 2374 2375 2376 2377 23770 23771 23772 23773 23779 2379 238 2380 2381 2382 2383 2384 2385 2386 2387 23871 23872 23873 23874 23875 23876 23877 23879 2388 2389 239 2390 2391 2392 2393 2394 2395 2396 2397 2398 23981 23989 2399.
